# Impaired Whole-Blood Polymorphonuclear Leukocyte Migration as a Possible Predictive Marker for Infections in Preterm Premature Rupture of Membranes

**DOI:** 10.1155/S1064744901000370

**Published:** 2001

**Authors:** Andreas Glasner, Gerd Egger, Raimund Winter

**Affiliations:** 1 Department of Obstetrics and Gynecology Univ. Frauenklinik–Graz Auenbruggerplatz 1 Graz A–8010 Austria; 2 Institute of Pathophysiology All Karl Franzens University Graz Austria

## Abstract

*Objectives:* Steroids, used in pretermpremature rupture of membranes (pPROM), to reduce the risk of morbidity and mortality of the preterm neonate, impair the maternal polymorphonuclear leukocyte (PMN)-based immune system. In spite of combination with antibiotics, prenatal and postnatal bacterial infections of mother and child are frequent. This pilot study focuses on the influence of steroids in pPROM on maternal PMN functional capacity and subsequent infections.

*Methods:* After opting for expectant management, eight women with pPROM and no signs of infection were treated by steroids (betamethasone 5.7 mg, i.m. every 24 hours, for three days) and antibiotic therapy with either amoxicillin and clavulanic acid, piperacillin or ampicillin i.v. up to delivery. The conventional inflammation parameters of PMN blood count and C-reactive protein (CRP) were measured daily in parallel with PMN migratory capacity towards N-formyl-methionyl-leucyI-phenylalanine stimulation and under blank conditions, estimated by a whole blood membrane filter assay.

*Results:* In all patients PMN migration decreased during the application of steroids. Three patients showed a decrease in PMN migration below critical values and in spite of antibiotic prophylaxis acute pyelonephritis developed 2–6 days later. PMN count and CRP were not predictive of maternal infection.

*Conclusion:* Reduced PMN function, caused by steroid treatment in pPROM, is suggested to be a reason for serious bacterial infections in spite of antibiotic prophylaxis. PMN migration reflects individual PMN defensive capacity.


*Infect Dis Obstet Gynecol 2001;9:227–232*

### Impaired whole-blood polymorphonuclear leukocyte migration as a possible predictive marker for infections in preterm premature rupture of membranes

**Andreas Glasner[1], Gerd Egger[2], Raimund Winter[1]**

[1]*Department of Obstetrics and Gynecology,*
[2]*Institute of Pathophysiology, All Karl Franzens University, Graz, Austria*

***Objectives:*** Steroids, used in preterm premature rupture of membranes (pPROM), to reduce the risk of morbidity and mortality of the preterm neonate, impair the maternal polymorphonuclear leukocyte (PMN)-based immune system. In spite of combination with antibiotics, prenatal and postnatal bacterial infections of mother and child are frequent. This pilot study focuses on the influence of steroids in pPROM on maternal PMN functional capacity and subsequent infections.

***Methods:*** After opting for expectant management, eight women with pPROM and no signs of infection were treated by steroids (betamethasone 5.7 mg, i.m. every 24 hours, for three days) and antibiotic therapy with either amoxicillin and clavulanic acid, piperacillin or ampicillin i.v. up to delivery. The conventional inflammation parameters of PMN blood count and C-reactive protein (CRP) were measured daily in parallel with PMN migratory capacity towards N-formyl-methionyl-leucyl-phenylalanine stimulation and under blank conditions, estimated by a whole blood membrane filter assay.

***Results:*** In all patients PMN migration decreased during the application of steroids. Three patients showed a decrease in PMN migration below critical values and in spite of antibiotic prophylaxis acute pyelonephritis developed 2–6 days later. PMN count and CRP were not predictive of maternal infection.

***Conclusion:*** Reduced PMN function, caused by steroid treatment in pPROM, is suggested to be a reason for serious bacterial infections in spite of antibiotic prophylaxis. PMN migration reflects individual PMN defensive capacity.

Key words: IMPAIRED PMN FUNCTION; pPROM STEROIDS

In preterm premature rupture of membranes (pPROM), bacterial and fungal infections are a major cause of maternal and fetal morbidity and mortality[1–4]. After rupture of the amniotic membrane, the opened amniotic sac facilitates the access of pathogens to the amniotic fluid and membrane. To reduce the risk of complications such as respiratory distress syndrome, intraventricular hemorrhage and mortality of the preterm neonate antenatal administration of corticoid drugs is widely used[3,5,6]. To prevent maternal and fetal infections, antibiotics are used in patients with pPROM[3,5–8]. Nonetheless, infections are frequent in mother and child[3,5–8].

Polymorphonuclear leukocytes (PMN) represent the first line of defense against bacterial and fungal pathogens[9,10]. For an effective host defense, the PMN-based immune system must be intact. In a series of clinical studies with various diseases, our working group demonstrated that PMN migration

Correspondence to: Andreas Glasner, MD, Department of Obstetrics and Gynecology, Univ. Frauenklinik–Graz, Auenbruggerplatz 1, A–8010 Graz, Austria, Europe. Email:dr.a.glasner@chello.at is a reliable marker for the effectiveness of the PMN-based immune system. With increasing duration and degree of impaired PMN migration, the risk of infection increased in parallel, eventually resulting in the clinical manifestation of an infection. In this way, it was possible to predict impending infections[11–15].

The aim of the present pilot study was to examine whether these experiences can be transferred to pPROM. In pPROM patients treated with steroids and antibiotics, PMN migration was measured daily until discharge from the hospital. To demonstrate the predictive relevance, the course of PMN migration was retrospectively correlated with the occurrence of infections. As a comparison, the conventional inflammation parameters PMN blood count and C-reactive protein (CRP) were measured in parallel.

### SUBJECTS AND METHODS

### Test subjects

Eight women with no signs of infection and pPROM were opted for expectant management. Differential blood count and assessment of clinical symptoms were done daily, starting with hospitalization immediately after pPROM until discharge. PROM was confirmed by speculum examination, AMNI-Check® (AMNI-Check, Lateral-Flow-Immunoassay, Mast-Diagnostica Laboratoriums-Präparate Gmbh, D-23858 Reinfeld) and sonography. Rupture of membranes continued until delivery.

Immediately after hospitalization for pPROM, 10 ml blood was taken from the cubital vein for determination within 20 min of PMN migration, leukocyte and differential blood counts and CRP. After opting for expectant management, we treated all eight women with 5.7 mg betamethasone i.m. every 24 h for 3 days (CELESTAN® biphase – ampoules, SP Labo, Heist, Belgien), combined with antibiotic prophylaxis, either amoxicillin and clavulanic acid i.v, piperacillin i.v. or ampicillin i.v. up to delivery. Blood samples were taken daily at 8 a.m.

Fetal heart rate was evaluated daily. Four preterm infants were delivered by Cesarean section because of non-reassuring fetal heart rate pattern, as indicated by declining fetal heart rate upon

**Table 1** Patient data and correlation to infection

| | *Patient* | | | | | | | |
|---|---|---|---|---|---|---|---|---|
| | *1* | *2* | *3* | *4* | *5* | *6* | *7* | *8* |
| GA – pPROM | 21 | 27 | 31 | 29 | 22 | 28 | 30 | 34 |
| GA – Del | 28 | 28 | 34 | 30 | 27 | 29 | 31 | 34 |
| Del – mod | c.s | c.s | c.s | c.s | sp | sp | sp | sp |
| Steroids | + | + | + | + | + | + | + | + |
| Antibiotics | A1 | A2 | P1 | P1 | P2 | A2 | A3 | S1 |
| TMI-FMLP < 6 | 3.8 | 2.3<br>1.2<br>0.9 | 1 | 1.4 | – | – | – | – |
| TMI-Q < 0,3 | 0.14<br>0.2 | 0.1<br>0.056<br>0.052 | 0.12 | – | – | – | – | – |
| Infection – Mother | + | + | + | – | – | – | – | – |
| Onset / Infection | 6 | 3 | 2 | | | | | |
| Infection – Newborn | + | – | + | – | – | – | – | – |

GA – pPROM, gestational age at pPROM; GA – Del, gestational age at delivery; Del – mod, delivery modus; c.s, Cesarean section; sp, spontaneous; Steroids, i.m. before delivery; Antibiotics, i.v. before delivery; A1, amoxicillin and clavulanic acid (2.2 g i.v. single shot); A2, amoxicillin and clavulanic acid (3 × 2.2 g i.v. 4 days); A3, amoxicillin and clavulanic acid (3 × 2.2 g i.v. 7 days); P1, piperacillin (2 × 4 g i.v. 13 days); P2, piperacillin (3 × 4 g i.v. 5 days); S1, ampicillin (3 × 2 g i.v. 3 days); TMI – FMLP, values of total migration indices under FMLP stimulation below the critical mark of '6'; TMI – Q, values of the total migration indices FMLP/blank quotients below the critical mark of '0.3'; Infection – Mother, Onset/Infection, Onset of maternal infection in days, after decreased TMI-FMLP and TMI-Q values; + yes; – no cardiotocography. Four preterm infants were delivered spontaneously and there was one stillbirth, due to prolapsed umbilical cord in the 27th week of pregnancy (Patient 5, Table 1). None of the eight women had any signs of infection or chorioamnionitis upon delivery. All patients gave written and verbal informed consent and the study was approved by the hospital's Ethics Committee.

### PMN blood count

Blood leukocyte count and differentiation/μl was done in a Coulter-Counter ZM.

### PMN migration

PMN migration was measured with a novel membrane filter migration assay. The main feature of this device is the use of fresh whole blood. In brief, 300 μl fresh whole blood anticoagulated with ammonium heparin (NH4 heparin syringes, Sarstedt, Nümbrecht, Germany) was diluted with HBSS 1:4 and processed 20 min after withdrawal without cell separation to avoid cell stimulation[14]. The migration device includes a cellulose nitrate membrane migration filter (3 μm pore size, 140 μm thick, Sartorius, Göttingen, Germany) and a chemoattractant depot containing the bacterial chemotactic peptide N-formyl-methionyl-leucyl-phenylalanine (FMLP) in solid form, and in parallel, blank chambers, each in triplicate. FMLP dissolves in the blood suspension pipetted into the chamber and forms a chemotactic gradient within the migration filter. The number of PMNs to penetrate into the migration filter is counted in consecutive layers of 10 μm throughout the entire depth of the filter. A count contains the number of immigrated PMNs and their distribution within the filter. In this study two relative PMN functions were considered: 'total migration

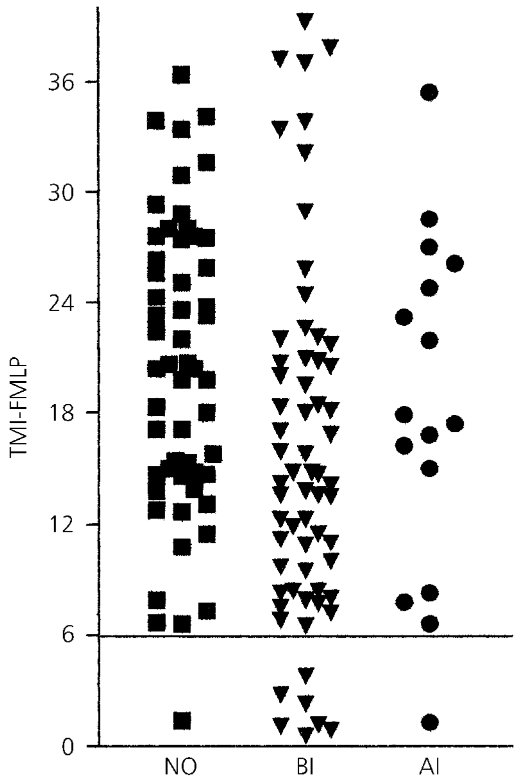

**Figure 1** Total migration index-FMLP (TMI-FMLP). Individual data of the five patients without infection (NO), and the three patients with infection: BI (before infection), AI (after onset of infection). The discriminative value of 6 is considered to be a risk factor and predictive for infection

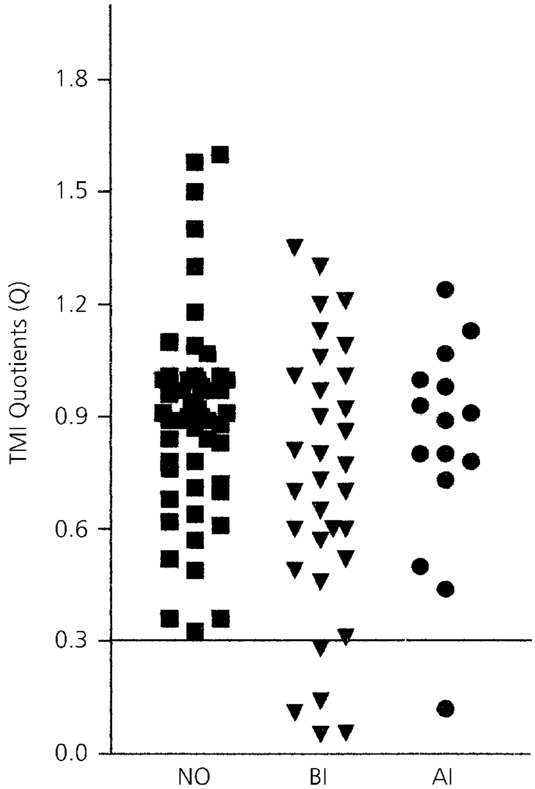

**Figure 2** Quotient TMI-FMLP/TMI-Blank (Q-TMI). Individual data of the five patients without infection (NO), and the three patients with infection: BI (before infection), AI (after onset of infection). The discriminative value of 0.3 is considered to be a risk factor and predictive for infection index' (TMI), i.e. the percentage of PMNs migrating into the filters upon FMLP stimulation and under blank conditions; the FMLP/Blank-quotient (Q), i.e. PMN reactivity to FMLP stimulation in comparison with the blanks. For more details about the method see article by Egger and colleagues[11].

### CRP

CRP was measured by Tina-quant® CRP (BM/Hitachi 704/705/71/911 SYS1, 1299859).

### Diagnosis of infections

Apparent maternal infection (pyelonephritis) was established via urine sediment, urine culture and clinical signs (nephralgia, temperature > 38°C).

Neonatal infection was confirmed by PMN band forms, increased CRP and clinical signs of centralization of circulation, hypotension, tachypnea, elevated temperature). All neonatals underwent chest X-ray to identify neonatal pneumonia. The causative microorganism in the newborn of patient 1 was found by wound smear and culture. In the newborn of patient 3 no causative microorganism could be found.

### Statistical analysis

The individual parameters TMI-FMLP, TMI-blank, the quotient Q, PMN blood counts and CRP were divided in three groups according to the clinical course of the patients: women without infection (NO) and women who developed infections; their values were separated into the groups before clinical manifestation of infection (BI) and after onset of infection (AI), and presented as scatter plots (Figures 1–3). The limits distinguishing an uneventful course from future infectious complications were established empirically by visual estimation and were chosen to achieve a minimal error rate.

### RESULTS

### Maternal infections

After decrease under the critical marks of TMI-Q < 0.3 and TMI-FMLP < 6 patients 1, 2

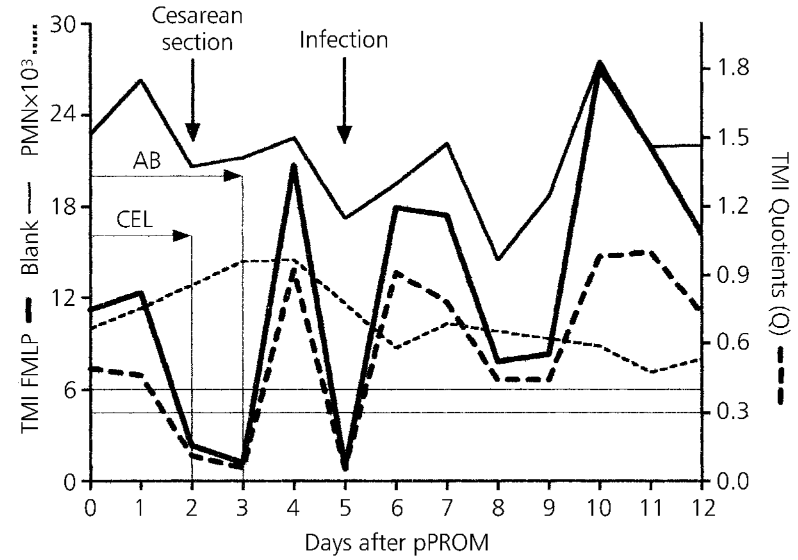

**Figure 3** Time profile of total migration indices (TMI), PMN blood counts/μl (PMN) and the duration of steroid therapy (CEL) and antibiotic prophylaxis (AB) of patient 2 (pyelonephritis). *Abscissa*: Days after pPROM are equal to days with monitoring. *Left ordinate:* TMI-FMLP (full stroke), the lower limit is 6. TMI-Blank (fine line). PMN blood counts (PMN, fine dotted fine). *Right ordinate*: The FMLP/Blank Quotient (Q, thick dotted line), the lower limit is 0.3. Note that during application of steroids (CEL) PMN functional ability (TMI-Q and TMI-FMLP) decreased very impressively. Infection (pyelonephritis) occurred three days later (day 5)

and 3 developed acute pyelonephritis 2–5 days later (Figures 1–3, Table 1). The microorganisms found in culture were: for Patient 1, *Escharichia coli*; Patient 2, *E. coli, Citrobacter diversus*; Patient 3, *E. coli, Klebsiella pneumoniae, Group D Streptococcus*. Patient 4 showed an isolated decrease of TMI-FMLP < 6, without subsequent infection. The PMN reactivity of patients 5, 6, 7, and 8, who did not develop infections, did not drop under the critical marks of TMI-Q < 0.3 and TMI-FMLP < 6.

### Neonatal infections

The newborn of patient 1 developed gangrene of the left lower leg; the causative microorganism was *Citrobacter* species. The newborn of patient 3 developed early onset sepsis. The six other newborns did not show any signs of neonatal infection. All neonatals underwent chest X-ray, none of them showed any sign towards neonatal pneumonia.

### PMN blood counts and CRP

The PMN blood counts/μl of patients 1, 2, 3 suffering from infections were within the range of those without infection (NO) (Figure 4).

During expectant management, serum CRP levels of all mothers were similar, between 0.1 up to 10 mg/l. Serum CRP levels of all mothers rapidly rose immediately after vaginal delivery or Caesarean section up to 20 mg/l[16–18] and, thus, masked the reaction to infection.

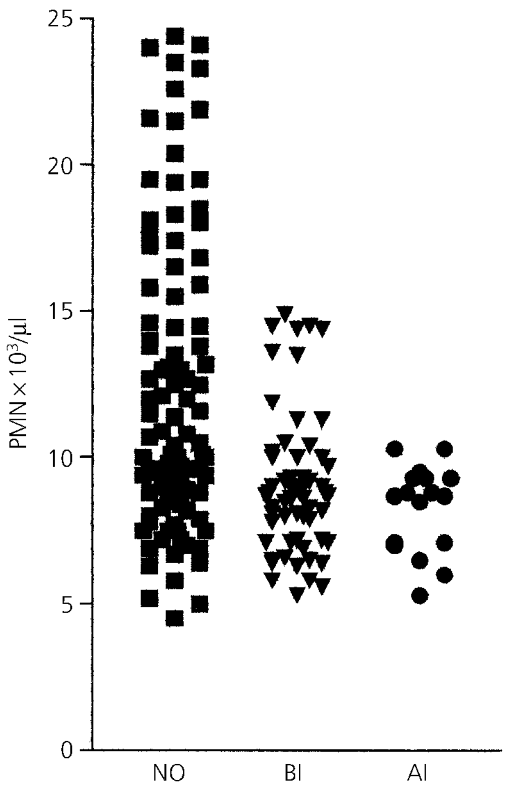

**Figure 4** PMN blood counts /μl. Individual PMN blood counts/μl of the five patients without infection (NO), and the three patients with infection: BI (before infection), AI (after onset of infection). The PMN blood counts/μl of patients before (BI) and after onset of infection (AI) are within the values of those patients without (NO) infections

### DISCUSSION

Preterm PROM increases maternal and fetal risk of infection and preterm labor complications[1–4]. Prenatal use of steroids and antibiotic prophylaxis are able to prolong the time between pPROM and delivery and to improve the initial condition of the newborn[1–8]. However, this therapy is applied without reliable knowledge of its effects on PMN function. PMNs play a crucial role in the host defense against bacterial and fungal pathogens[9,10] and the maternal PMN functional state can be hampered by steroid treatment.

This first approach to the problem 'definition of the infection risk in pPROM' suggests that an impairment of PMN migratory efficacy is a predictive marker for infection, and that in an organism with an impaired immune defence, infections can develop despite antibiotic prophylaxis. Our data are in good agreement with earlier studies by our working group on other topics demonstrating that the mere number of circulating PMNs does not necessarily reflect their immunological efficiency, and functional parameters are much more reliable. Before infections, we often found a low PMN readiness to migrate that coincided with elevated blood counts feigning an effective host defense[11–15].

We conclude that the conventional marker for PMN efficiency is not reliable. However, the value of PMN migration in pPROM and perinatal infections has to be consolidated in more comprehensive studies.

### REFERENCES

1. Goldenberg RL, Rouse DJ. Prevention of premature birth. *New Engl J Med* 1998;339:313–20
2. Mercer BM, Arheart KL. Antimicrobial therapy in expectant management of preterm rupture of the membranes. *Lancet* 1995;346:1271–9
3. Ohlsson A. Treatments of preterm premature rupture of the membranes: A meta-analysis. *Am J Obstet Gynecol* 1989;160:890–8
4. Strittmatter HJ, Wischnik A, Lasch P, *et al.* Fetal outcome bei Frühgeborenen unter 1500g Geburtsgewicht unter besonderer Berücksichtigung des Surfactantbedarfs. *Geburtshilfe Frauenheilkd* 1992; 52:544–8
5. Mercer BM, Moretti ML, Prevost RR, Sibai BM. Erythromycin therapy in preterm premature rupture of the membranes: a prospective, randomized trial of 220 patients. *Am J Obstet Gynecol* 1992; 166:794–802
6. Crowley PA. Antenatal corticosteroid therapy: a meta-analysis of the randomized trials, 1972 to 1994. *Am J Obstet Gynecol* 1995;173:322–35
7. Lewis DF, Brody K, Edwards MS, *et al.* Preterm premature ruptured membranes: a randomized trial of steroids after treatment with antibiotics. *Obstet Gynecol* 1996;88:801–5
8. Mercer BM, Miodovnik M, Thurnau GR, *et al.* Antibiotic therapy for reduction of infant morbidity after preterm premature rupture of the membranes: a randomized controlled trial. *J Am Med Assoc* 1997;278:989–95
9. Edwards SW. *Biochemistry and Physiology of the Neutrophil.* Cambridge University Press: 1994:1–32
10. Holland SM, Gallin JI. Disorders of phagocytic cells. In Gallin JI, Snyderman R eds. *Inflammation: Basic Principles and Clinical Correlates.* 3rd edition. Philadelphia: Lippincott Williams & Wilkins, 1999:910
11. Egger G, Klemt CH, Spendel S, *et al.* Migratory activity of blood polymorphonuclear leukocytes during juvenile rheumatoid arthritis, demonstrated with a new whole-blood membrane filter assay. *Inflammation* 1994;18:427–41
12. Egger G, Burda A, Hengster P, *et al.* Polymorphonuclear leukocyte functions as predictive markers for infections after organ transplantation. *Transpl Int* 2000;13:114–21
13. Egger G, Allmayer T, Iberer F, *et al.* Impaired polymorphonuclear leukocyte migration as a possible monitor for the risk of septicaemia in immunosuppressed transplant patients. *Med Sci Res* 1996;24: 17–19
14. Egger G, Kukovetz EM, Hayn M, Fabjan JS. Changes in the polymorphonuclear leukocyte function of blood samples by storage time, temperature and agitation. *J Immunol Meth* 1997;206: 61–71
15. Hofer HP, Kukovetz EM, Egger G, *et al.* PMN-related parameters for the monitoring of wound healing traumatology. *Eur J Orthop Surg Traumatol* 1995;5:21–6
16. Keski-Nisula L, Kirkinen P, Ollikainen M, Saarikoski S. C-reactive protein in uncomplicated parturients delivered by caesarean section. *Acta Obstet Gynecol Scand* 1997;76:826–7
17. Staven P, Suonio S, Saarikoski S, Kauhanan O. C-reactive protein (CRP) levels after normal and complicated caesarean section. *Ann Chir Gynaecol* 1989;78:142–5
18. Romem Y, Artal R. C-reactive protein in pregnancy and in the postpartum period. *Am J Obstet Gynecol* 1985;151:380–3

RECEIVED 05/23/01; ACCEPTED 08/27/01
